# Intratympanic Versus Systemic Steroid Therapy for Idiopathic Sudden Hearing Loss: A Systematic Review and Meta-Analysis

**DOI:** 10.7759/cureus.22887

**Published:** 2022-03-06

**Authors:** Christos Sialakis, Christos Iliadis, Aikaterini Frantzana, Petros Ouzounakis, Lambrini Kourkouta

**Affiliations:** 1 Department of Otolaryngology, General Hospital “Agios Dimitrios-G. Gennimatas”, Thessaloniki, GRC; 2 Department of Nuclear Medicine, Private Diagnostic Health Center, Thessaloniki, GRC; 3 School of Health Sciences, European University Cyprus, Thessaloniki, GRC; 4 Nursing, General Hospital of Alexandroupoli, Alexandroupoli, GRC; 5 Nursing, International Hellenic University, Thessaloniki, GRC

**Keywords:** transtympanic steroid treatment, deafness, methylprednisolone, dexamethasone, oral steroid treatment, idiopathic hearing loss, randomized controlled trials, systemic steroid therapy, sudden hearing loss, intratympanic steroid therapy

## Abstract

Idiopathic sudden sensorineural hearing loss (ISSHL) is a common condition characterized by hearing threshold reduction, most often of unknown causes. The patient experiences a sudden reduction of the hearing threshold in one or both ears. Steroids are the mainstream of the treatment. This study aims to investigate the effectiveness of intratympanic steroid administration compared with systemic administration and the combination of the two steroid treatments in the hearing recovery of patients with idiopathic sudden sensorineural hearing loss. We searched electronic databases such as PubMed, ScienceDirect, CINAHL, Cochrane (Central), Ovid, and Medline from August 31, 2021, to November 31, 2021, and from February 5 to 10, 2022. We included 12 randomized controlled trials (RCTs) and performed a meta-analysis comparing the efficiency in the hearing recovery of intratympanic versus systemic steroid treatment, systemic versus combined, and intratympanic versus combined steroid treatment. The results of the intratympanic versus systemic steroid therapy comparison showed no actual difference in efficiency and no statistical significance (odds ratio: 1.07 (Mantel-Haenszel (M-H), fixed, 95% confidence interval (CI): 0.76-1.51)). Systemic steroid treatment was inferior to combined steroid treatment and was the only outcome with statistical significance (odds ratio: 0.55 (M-H, fixed, 95% CI: 0.38-0.80)). Intratympanic steroid treatment was inferior to combined steroid treatment, although the results were not statistically significant (odds ratio: 0.65 (M-H, fixed, 95% CI: 0.37-1.16)). In conclusion, systemic steroid therapy was inferior to combined steroid therapy. The comparison of intratympanic with systemic therapy and intratympanic with combined therapy showed no statistical significance. Further research is needed with more RCTs, and side effects should be considered.

## Introduction and background

Idiopathic sudden sensorineural hearing loss (ISSHL) is defined as a rapid decline in hearing for three days or less affecting three or more frequencies by 30 dB or more without any identifiable cause [1]. In the United States, the incidence is between 5 and 27 per 100,000 people and 60,000 new cases per year [2]. Although SSHL shows a tendency toward spontaneous hearing recovery, steroids are widely used in practice [3]. Although intratympanic steroids offer a choice in primary care, it is not conclusive if they are better or worse than the traditional treatment option [4]. The first report of the use of intratympanic steroids for sudden SNHL was by Silverstein et al. in 1996 [5]. According to this study, dexamethasone (DXM), when placed in the middle ear, diffuses into the inner ear and produces an effect on the cochlea in certain patients.

This study aims to investigate the effectiveness of intratympanic steroid therapy (ITS) compared with systemic steroid therapy and the efficiency of the combination of treatments in patients with ISSHL. We performed a review of RCT studies in English literature and proceed to meta-analysis.

## Review

Methods

This study adheres to the guidelines of the Preferred Reporting Items for Systematic Reviews and Meta-Analysis (PRISMA) 2020 checklist [6].

Inclusion and Exclusion Criteria

We used the keywords “intratympanic steroid therapy,” “sudden hearing loss,” “systemic steroid therapy,” “randomized controlled trials,” “idiopathic hearing loss,” “transtympanic steroid treatment,” “oral steroid treatment,” “dexamethasone,” “methylprednisolone,” “deafness,” and “corticosteroids.” The inclusion criteria were set as follows: the studies must be randomized controlled trials, the objectives of the studies must be the efficiency of intratympanic steroid therapy (ITS) and systemic steroid therapy (SST) for patients affected by SSHL, and the studies should be in English. The exclusion criteria were set as follows: studies that were not RCTs, studies that compare therapies other than steroids, and studies that compare steroid therapy to treat ear diseases other than ISSHL.

Information Resources and Search Strategy

Electronic search on PubMed, ScienceDirect, CINAHL, Cochrane (Central), Ovid, and Medline from August 31, 2021, to November 31, 2021, and from February 5 to 10, 2022, was performed to identify studies that meet the inclusion criteria.

Selection Process and Data Collection Process

One of the authors first screened the abstracts of the collected studies independently, and after that, another author screened the abstracts. Any disagreement was resolved by discussion. Data collected were about the method of steroid administration, size of study arms, year of the publication of the study, outcomes of the study, numbers of patients who recovered in each study arm, age range of patients in each study, definition of criteria for hearing recovery, and follow-up time. The authors worked independently during the data collection process.

Effect Measures

In this review, we measured three outcomes: first, the effect of intratympanic steroid therapy versus systemic steroid therapy; second, systemic steroid therapy versus combined therapy (intratympanic and systemic therapy); and third, intratympanic steroid therapy versus combined therapy. For the effect measures, the odds ratio was preferred using the Mantel-Haenszel (M-H) analysis calculating 95% confidence intervals (95% CIs), with fixed effect.

Evaluation of the Quality of the Included Studies

To evaluate the quality of the studies, two authors independently used the Cochrane Collaboration Tool for Assessing Risk of Bias [7] and the Review Manager (RevMan) 5.4 software package [8]. According to these guidelines, the key points to evaluate the risk of bias in each of the included studies were random sequence generation (selection bias), allocation concealment (selection bias), blinding of participants and personnel (performance bias), incomplete outcome data (attrition bias), selective reporting (reporting bias), and other bias. The evaluation is based on three options, either low, high, or unclear risk of bias for each element.

Statistical Methods

In this review, the RevMan 5.4.1 [8] package software was used to perform the meta-analysis. Statistical processing was performed based on the data extracted from the included RCTs, comparing the subgroups of each study (intratympanic steroid therapy, systemic steroid therapy, or combined steroid therapy), and for this reason, the dichotomous option was selected. In this meta-analysis, the odds ratio fixed effect was preferred for each outcome. Furthermore, heterogenicity I and test for overall effect Z was calculated by the software. Heterogenicity I2 was considered as follows: 0%-40%, not important; 40%-75%, moderate degree heterogenicity; and 75%-100%, considerable degree of heterogenicity.

Results

**Figure 1:**
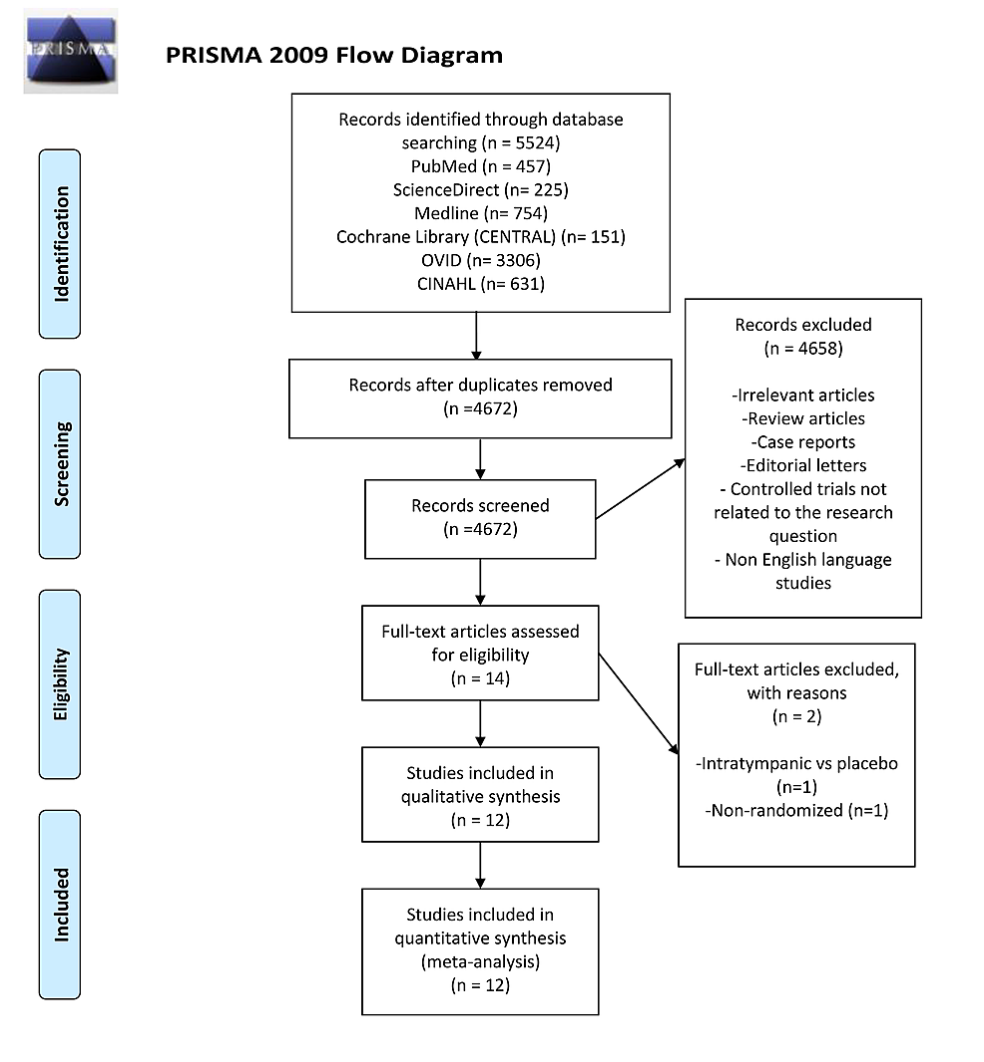
Flowchart of the process of study selection PRISMA: Preferred Reporting Items for Systematic Reviews and Meta-Analysis [9]

In this meta-analysis, nine studies met the inclusion criteria. A flow diagram that shows the screening process and study selection is presented in Figure 1.

Table 1 presents the characteristics of each of the selected studies [10-21] included in the study.

**Table 1:** Characteristics of each of the selected studies RCTs: randomized controlled trials; ITS: intratympanic steroid therapy; SST: systemic steroid therapy; PTA: pure tone average; SNHL: sensorineural hearing loss; ISNHL: idiopathic sensorineural hearing loss

Study	Treatment protocol and follow-up time	RCT subgroup participants and mean age
Dispenza et al. (2011) [10]	Intratympanic: transtympanic injection solution of dexamethasone 4 mg/mL weekly for a total of four injections; systemic: oral 60 mg of prednisone tapered over 14 days; follow-up time: six months	Intratympanic: 25; systemic: 21. Intratympanic: 47 years; systemic: 54 years
Ermutlu et al. (2018) [11]	Intratympanic: 0.5–0.7 cc dexamethasone (DXM) (8 mg/2 mL); systemic: oral prednisolone 1 mg/kg (maximum: 80 mg) and tapering 10 mg every three days; follow-up time: three months	Intratympanic: 19; systemic: 16. Intratympanic: 49.68 years; systemic: 41.06 years
Kosyakov et al. (2011) [12]	Intratympanic: dexamethasone 4 mg/cc every day for 10 days, 4 mg every other day for 20 days, and 4 mg twice a week for five months; systemic: dexamethasone (0.1 mg/kg) in 200 mL of isotonic solution intravenously over 10 days; follow-up time: six months	Intratympanic: 25; systemic: 25. Intratympanic: 49 years; systemic: 40 years
Ashtiani et al. (2018) [13]	Intratympanic: 0.6 mL vials of methylprednisolone on days 1, 5, 9, and 13; systemic: oral 75 mg/day prednisolone over 10 days; intratympanic + systemic: 0.6 mL vials of methylprednisolone + oral 75 mg/day prednisolone over 10 days; follow-up time: four weeks	Intratympanic: 32; systemic: 45; intratympanic + systemic: 35. Intratympanic: 48.69 years; systemic: 43.59 years; intratympanic + systemic: 40.8 years
Battaglia et al. (2008) [14]	Intratympanic: 0.5–0.7 mL of intratympanic dexamethasone (12 mg/mL) once a week for three weeks; systemic: 60 mg oral prednisone for seven days, 50 mg for two days, and 40 mg for two days, and then 10 mg until finished for three weeks; intratympanic + systemic: combination of treatment as above; follow-up time: three months	Intratympanic: 17; systemic: 18; intratympanic + systemic: 16. Intratympanic: 60 years; systemic: 54 years; intratympanic + systemic: 57 years
Lee et al. (2011) [15]	Systemic: oral steroids (60 mg/day for five days, followed by tapering for five days; intratympanic + systemic: (within two days) after initial steroid therapy; intratympanic: dexamethasone disodium phosphate, 5 mg/mL in the amount of 0.3–0.4 mL twice a week for two consecutive weeks; follow-up time: six weeks	Systemic: 25; intratympanic + systemic: 21. Systemic: 44.0 ± 16.2 years; intratympanic + systemic: 45.3 ± 13.5 years
Ahn et al. (2008) [16]	Systemic: 14-day course of oral steroids (48 mg methylprednisolone for nine days, followed by tapering for five days); intratympanic + systemic: intratympanic 0.3–0.4 mL of 5 mg/mL dexamethasone on days 1, 3, and 5 + 14-day course of oral steroids (48 mg methylprednisolone for nine days, followed by tapering for five days); follow-up time: three months	Intratympanic: 60; intratympanic + systemic: 60. Intratympanic: 48.6 ± 15.4 years; intratympanic + systemic: 45.9 ± 14.7 years
Arastou et al. (2013) [17]	Systemic: oral prednisolone (1 mg/kg/day for 10 days); intratympanic + systemic: oral prednisolone (1 mg/kg/day for 10 days) + intratympanic dexamethasone 0.4 mL of 4 mg/mL twice a week for two weeks; follow-up time: two weeks	Systemic: 36; systemic + intratympanic: 36. Systemic: 49.17 years; systemic + intratympanic: 45.4 years
Lim et al. (2013) [18]	Intratympanic: 0.3–0.4 mL intratympanic dexamethasone (5 mg/mL) twice a week for two weeks; systemic: 60 mg oral prednisolone for five days, 40 mg for two days, 20 mg for two days, and 10 mg for one day; intratympanic + systemic: intratympanic treatment as described while simultaneously taking an oral steroid for two weeks; follow-up time: three weeks	Intratympanic: 20; systemic: 20; intratympanic + systemic: 20. Intratympanic: 53.3 ± 15.3 years; systemic: 51.3 ± 14.5 years; intratympanic + systemic: 47.8 ± 14.2 years
Rauch et al. (2011) [19]	Intratympanic: 1 mL doses of 40 mg/mL of methylprednisolone over two weeks every 3–4 days; systemic: 60 mg/day prednisone for 14 days, followed by a five-day taper (50 mg, 40 mg, 30 mg, 20 mg, and 10 mg), for a total of 19 days of treatment; follow-up time: six months	Intratympanic: 129; systemic: 121. Intratympanic: 51.3 years; systemic: 50.4 years
Tsounis et al. (2018) [20]	Intratympanic: 0.4–0.6 mL of 62.5 mg/mL methylprednisolone on three, five, and 10 days after the presentation (a total of four times); systemic: intravenous prednisolone 1 mg/kg for seven days, followed by 0.5 mg/kg per day for three days, followed by oral methylprednisolone 32 mg/day for four days and oral methylprednisolone 16 mg/day for another three days; intratympanic + systemic: combination as above; follow-up time: three months	Intratympanic: 34; systemic: 35; combined: 33. Intratympanic: 53.2 ± 12 years; systemic: 50.1 ± 17.3 years; intratympanic + systemic: 51.7 ± 15.8 years
Hong et al. (2009) [21]	Intratympanic: dexamethasone 0.3–0.4 cc (5 mg/mL) once a day for eight days; systemic: 60 mg oral prednisolone for four days, followed by 40 mg for two days and 20 mg for two days; follow-up time: three months	Intratympanic: 32; systemic: 31. Mean age: 56.9 years

Forests plots were created to show statistical analysis for the effect measures, heterogenicity, and test for overall effect. Moreover, funnel plots were created using the software to assess visually any publication bias. Odds ratios were calculated using Mantel-Haenszel statistical method [22].

Intratympanic Steroid Therapy Versus Systemic Steroid Therapy

**Figure 2:**
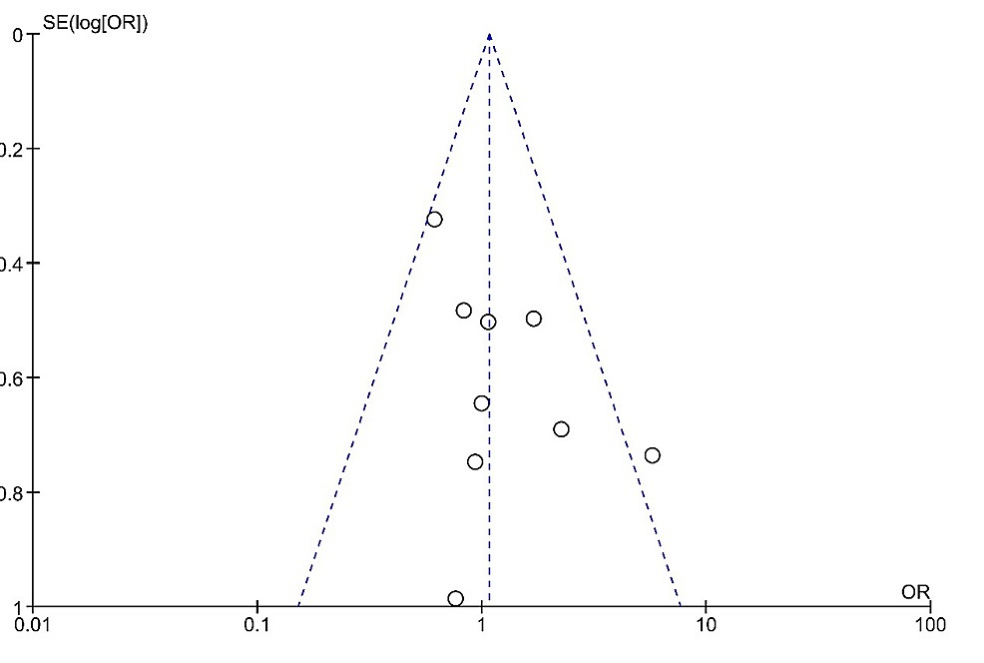
Funnel plot for the comparison of intratympanic steroid therapy versus systemic steroid therapy The funnel plot is symmetrical, indicating low publication bias.

**Figure 3:**
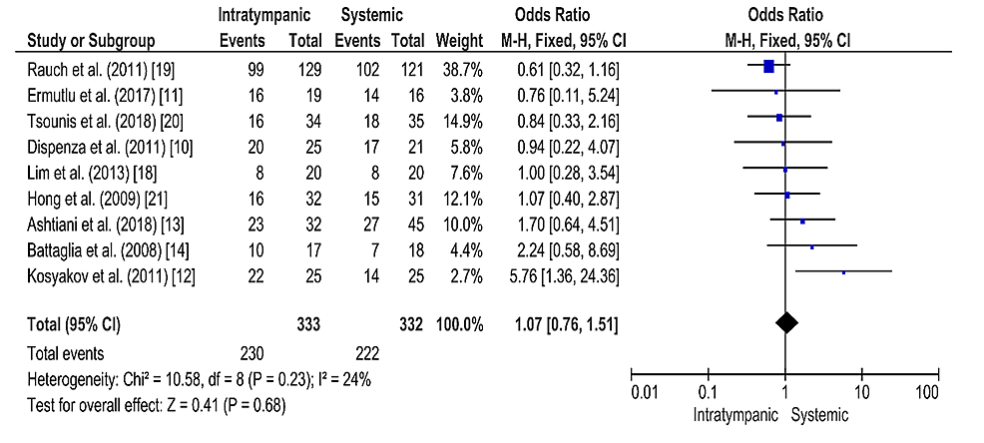
Forest plot for the comparison of intratympanic steroid therapy versus systemic steroid therapy

The funnel plot for intratympanic steroid therapy versus systemic steroid therapy, which is shown in Figure 2, is symmetrical, indicating low publication bias. The results of the forest plot are shown in Figure 3. In this comparison, nine randomized controlled trials were included. The results show 230 hearing recovery events from the total population of 333 patients of intratympanic intervention and 222 hearing recovery events from 332 patients of systemic intervention. The Mantel-Haenszel method was used. The odds ratio was 1.07, and the 95% confidence interval (95% CI) was 0.76-1.51. There is no statistically significant difference in the effectiveness of intratympanic therapy compared with systemic therapy, as the 95% confidence intervals contain the 1. The heterogenicity I2 was 24%. The test for overall effect Z was 0.41 (P = 0.68).

Systemic Steroid Therapy Versus Combined Steroid Therapy

**Figure 4:**
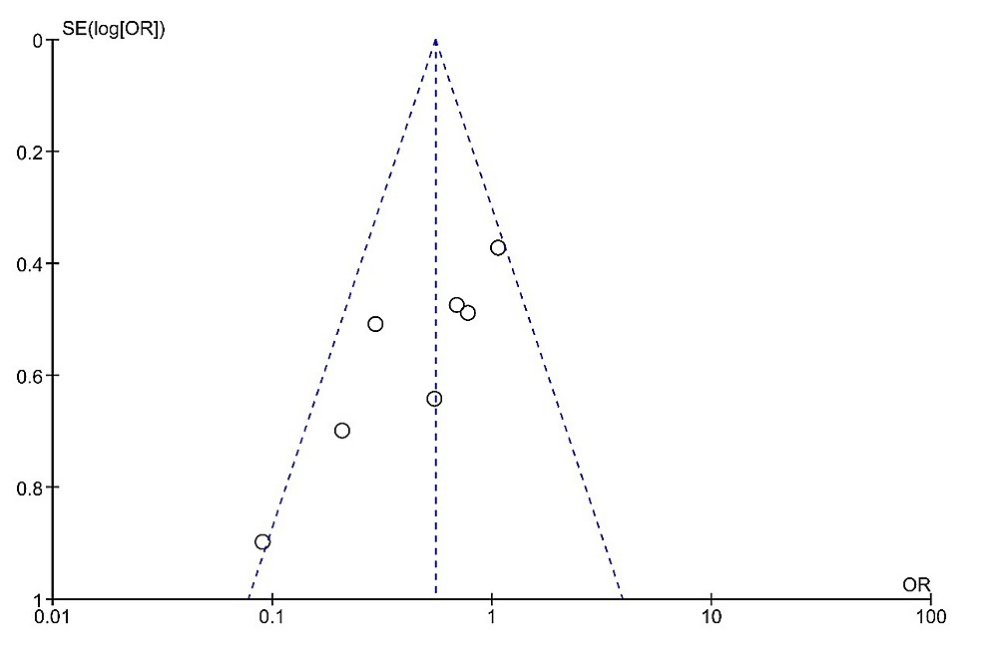
Funnel plot for the comparison of systemic steroid therapy versus combined steroid therapy The funnel plot is symmetrical, indicating low publication bias.

**Figure 5:**
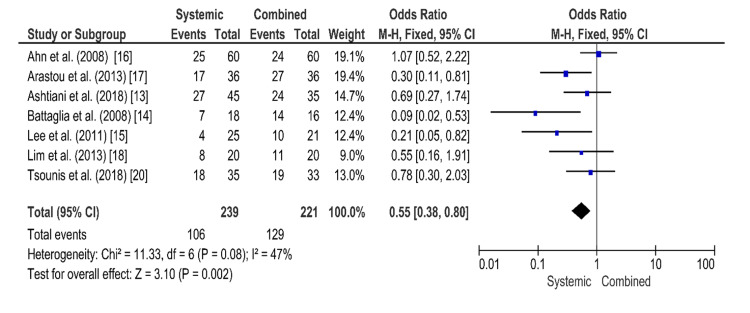
Forest plot for the comparison of systemic steroid therapy versus combined steroid therapy

The funnel plot for systemic steroid therapy versus combined steroid therapy, which is shown in Figure 4, is symmetrical, indicating low publication bias. The results of the forest plot are shown in Figure 5. In this comparison, seven randomized controlled trials were included. The results show 106 hearing recovery events from the total population of 239 patients of systemic steroid intervention and 129 hearing recovery events from the total population of 221 patients of combined steroid intervention. The Mantel-Haenszel method was used. The odds ratio was 0.55, and the 95% confidence interval (95% CI) was 0.38-0.80. There is a statistically significant difference in the effectiveness of the compared interventions. The results show a superiority of the combined steroid treatment. The heterogenicity I2 was 47%. The test for overall effect Z was 3.10 (P = 0.002).

Intratympanic Steroid Therapy Versus Combined Steroid Therapy

**Figure 6:**
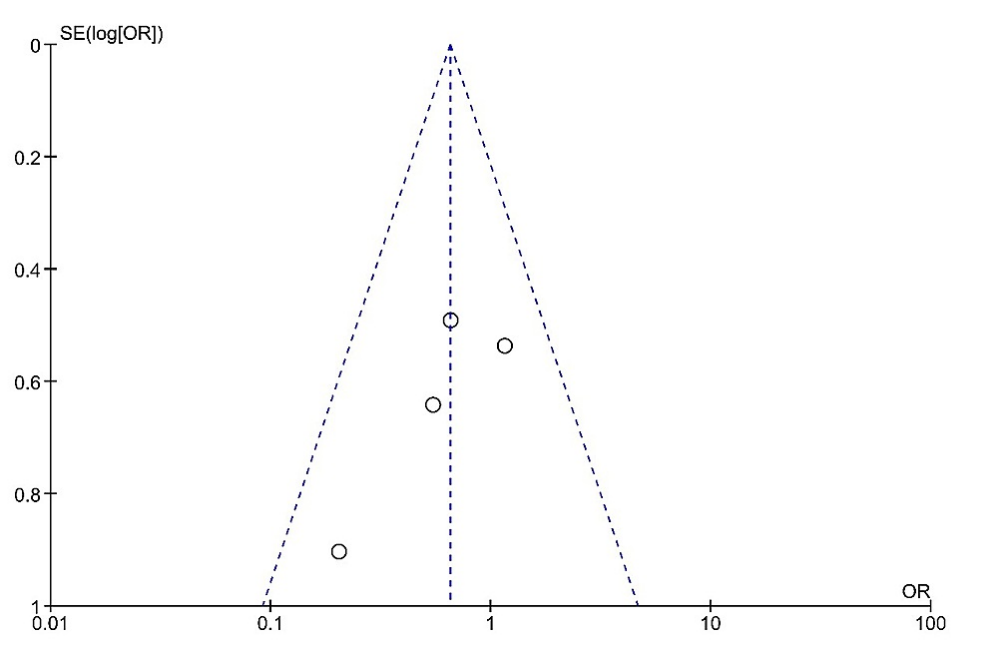
Funnel plot for the comparison of intratympanic steroid therapy versus combined steroid therapy The funnel plot is symmetrical, indicating low publication bias.

**Figure 7:**
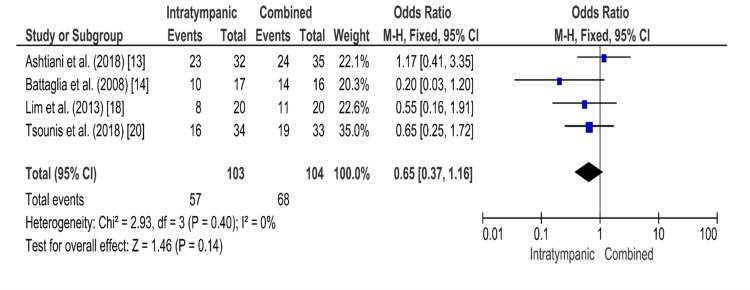
Forest plot for the comparison of intratympanic steroid therapy versus combined steroid therapy

The funnel plot for systemic steroid therapy versus combined steroid therapy, which is shown in Figure 6, is symmetrical, indicating low publication bias. The results of the forest plot are shown in Figure 7. In this comparison, four randomized controlled trials were included. The results show 57 hearing recovery events from the total population of 103 patients of the intratympanic steroid intervention and 68 hearing recovery events from the total population of 104 patients of combined steroid intervention. The Mantel-Haenszel method was used. The odds ratio was 0.65, and the 95% confidence interval (95% CI) was 0.37-1.16. There is no statistically significant difference in the effectiveness of intratympanic steroid therapy compared with combined steroid therapy, as the 95% confidence intervals contain the 1. The heterogenicity I2 was 0%. The test for overall effect Z was 1.46 (P = 0.14).

Risk of Bias Assessment

**Figure 8:**
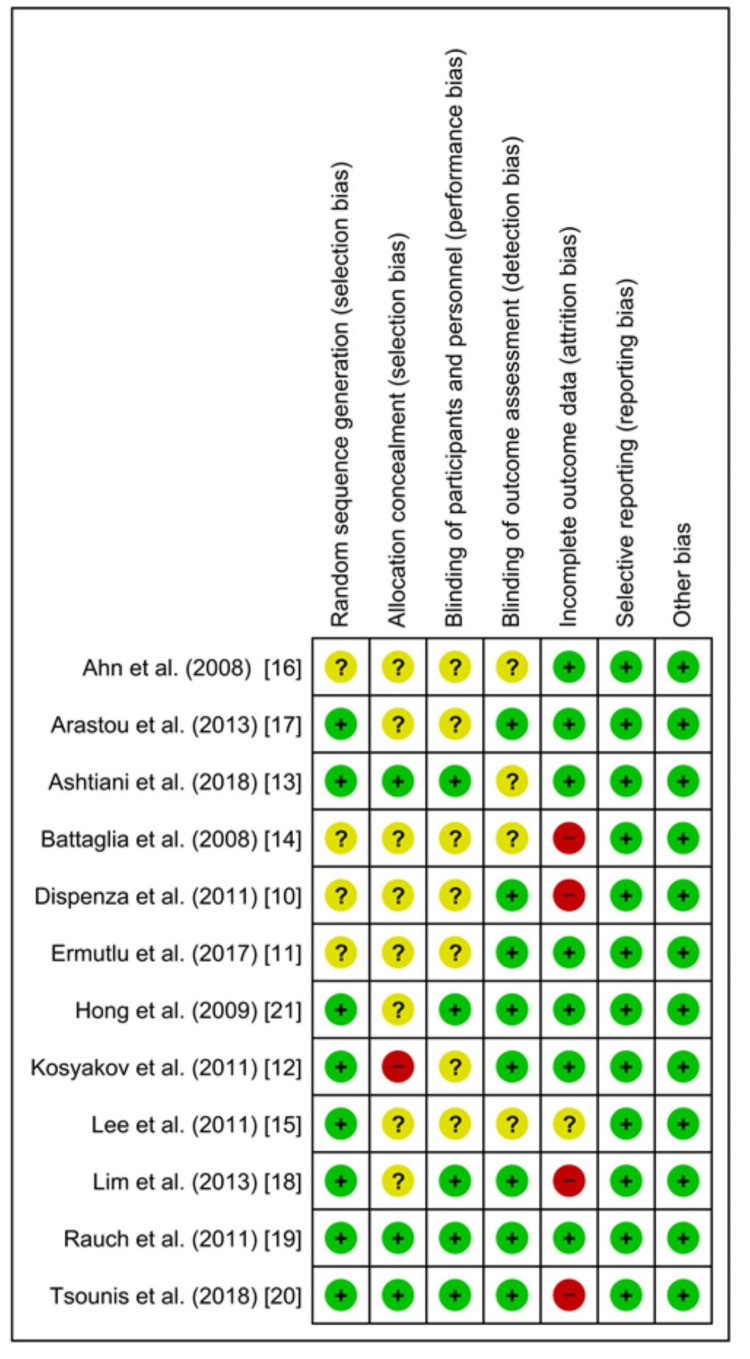
Risk of bias summary

**Figure 9:**
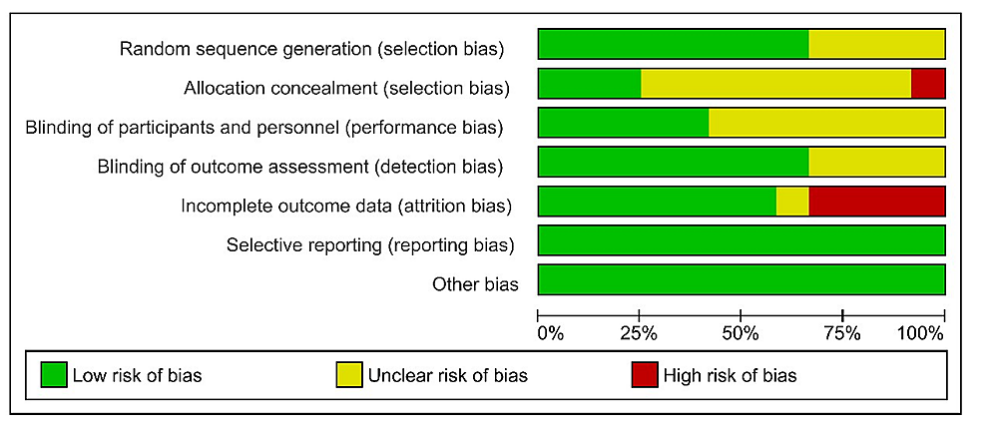
Risk of bias graph

According to the risk of bias assessment, which is shown in Figure 8 and Figure 9, none of the studies were detected with selective reporting (reporting bias). In four studies (Battaglia et al. [14], Dispenza et al. [10], Lim et al. [18], and Tsounis et al. [20]), a high risk of attrition bias was observed due to incomplete data outcome. Lee et al. [15] showed unclear information, and the rest of the studies showed a low risk of attrition bias. For the blinding of outcome assessment, Ahn et al. [16], Ashtiani et al. [13], Battaglia et al. [14], and Lee et al. [15] showed unclear information. The rest of the studies showed a low risk of detection bias. For the blinding of participants and personnel, seven studies showed unclear information (Ahn et al. [16], Arastou et al. [17], Battaglia et al. [14], Dispenza et al. [10], Ermutlu et al. [11], Kosyakov et al. [12], and Lee et al. [15]). The rest of the five studies were detected with a low performance bias. For allocation concealment, Kosyakov et al. [12] was detected with a high risk of bias. In the studies of Ashtiani et al. [13], Rauch et al. [19], and Tsounis et al. [20], a low risk of bias was observed. The rest of the studies presented unclear information. For random sequence generation, Ahn et al. [16], Battaglia et al. [14], Dispenza et al. [10], and Ermutlu et al. [11] showed unclear information. The rest of the studies were observed with a low risk of bias.

Discussion

In this meta-analysis, we included 12 RCTs, and we searched the electronic databases PubMed, ScienceDirect, CINAHL, Cochrane (Central), Ovid, and Medline Complete from August 31, 2021, to November 31, 2021, and from February 5 to 10, 2022. We searched English language studies and conducted a systematic review and meta-analysis following the PRISMA guidelines, and we assessed the risk of bias for each study. In this study, three comparisons were performed: intratympanic versus systemic steroid therapy, systemic versus combined steroid therapy, and intratympanic versus combined steroid therapy.

Different treatment protocols have been used from the included studies [10-21], either for intratympanic treatment or for systemic treatment. This heterogenicity may affect our results. We observed no statistically significant results in the comparison of intratympanic with systemic and in comparison of intratympanic with combined treatment. Combined treatment was superior to systemic treatment alone, and the results were statistically significant. The population of all comparison groups was well-matched: 333 patients of intratympanic to 332 patients of systemic treatment, 239 patients of systemic to 221 patients of combined treatment, and 103 patients of intratympanic to 104 patients of systemic treatment. Dexamethasone was the steroid of choice in most cases for the intratympanic administration, and for systemic administration, prednisone or prednisolone was mostly used.

Systemic steroid therapy is the main treatment option for idiopathic sudden sensorineural hearing loss, but intratympanic steroid administration may be used as initial treatment without systemic steroid administration, as simultaneous treatment with systemic steroids, or finally, as a salvage treatment in case systemic steroid therapy fails [17]. The mechanism of action of steroids in the inner ear is not yet clear, although intratympanic steroids offer the advantage of avoiding systemic side effects [16]. Several treatments for idiopathic sudden sensorineural hearing loss have been tested, such as *Ginkgo biloba*, magnesium, hyperbaric oxygen, and vasodilatory agents, although they were not effective [14]. Several adverse events have been reported for either systemic or intratympanic steroid treatment. Such adverse events are ear pain, tympanic membrane perforation, injection pain, mood change, dizziness or vertigo, dry mouth and thirst, blond glucose change, ear infection, weight change, appetite change, and sleep change [19].

Several systematic reviews with meta-analysis exist in the literature about the research question. Yang et al. [23] concluded that the treatment of SSHL has better efficiency with intratympanic steroids than systemic therapy, although further research is needed to confirm the results. Furthermore, the authors concluded that intratympanic steroid therapy has a higher cost due to multiple visits of the patient needed for treatment. Finally, the patient should be well informed about the advantages and disadvantages of each treatment option. Zhao et al. [24] concluded that patients who were treated with intratympanic steroid therapy showed statistically significant improvement compared with those treated with systematic steroid therapy, and the adverse effect that may arise are less frequent compared with systemic therapy. Qiang et al. [25] stated that intratympanic steroid therapy showed better results in pure tone average (PTA) than systemic steroid therapy, although the severity of hearing loss may influence the improvement and recovery of hearing, and thus, further research is needed. According to Li and Ding [26], there was no significant recovery rate comparing intratympanic and steroid therapy; instead, combination steroid therapy showed significant improvement compared with systemic steroid therapy. Moderate and high doses of combination therapy could accelerate the hearing recovery of patients. According to Garavello et al. [27], intratympanic steroid therapy is effective for treating SSHL as a salvage treatment, but not as a primary treatment. Further research is needed to confirm the findings according to the characteristics of patients and treatment protocols used. In contrast, Mirian and Ovesen [28] concluded that there was no difference in therapeutic outcomes in therapeutic comparisons between intratympanic, systemic, and combined steroid therapies. Moreover, Ng et al. [29] stated that intratympanic steroid therapy shows statistically significant improvement as a salvage treatment for patients who failed initially in systemic steroid therapy. El Sabbagh et al. [30] stated that there is not sufficient evidence to support intratympanic steroid therapy and alternative therapy.

Limitations of the Evidence Included and the Review Process

Despite the comprehensive research, this study has some limitations. First, different treatment protocols were applied for intratympanic and systemic treatment in each study. This may also influence the results and the ratio of patients’ recovery. Second, the health background of patients who participated in the studies may differ in some cases, although the demographics were similar in RCT subgroups. Third, some treatment protocols included in addition to steroid therapy are other medications that may have an unspecified impact on the treatment result and recovery rate of patients. Although we performed comprehensive research in electronic databases, we included only studies in English literature. Some studies were written in other languages, so they were not included in this meta-analysis. Furthermore, to our best research strategy, we searched several electronic databases, but not of all of them, such as the Embase database.

Implications of the Results for Practice, Policy, and Future Research

Our results from this review show the superiority of combination steroid therapy for the treatment of patients with idiopathic sensorineural hearing loss. We advanced our research beyond the comparison of intratympanic versus systemic steroid therapy. Considering as a strength of this study, we compared also intratympanic with combined steroid therapy. This review shows an integrated analysis of all outcomes investigated, as we performed a statistical analysis of comparisons of all treatments between them.

We suggest the standardization of treatment protocols for intratympanic steroid therapy and systemic steroid therapy, as well as better observation of the additional medications combined with steroid therapy. We suggest, in case other medications are added, that a different outcome from steroid monotherapy should be considered.

In the case of partial recovery, quality of life (QoL) and patient satisfaction should be investigated as future research.

## Conclusions

In this study, we compared the efficiency of intratympanic, systemic, and combined steroid therapy for patients with idiopathic sudden sensorineural hearing loss. According to the analysis performed, systemic steroid therapy was inferior to combined steroid therapy. The comparison of intratympanic with systemic therapy and intratympanic with combined therapy showed no statistical significance. Further research is needed with more RCTs, and side effects should be considered.
